# Umbilical Cord Mesenchymal Stem Cells Overexpressing Heme Oxygenase-1 Promotes Symptoms Recovery in Cystitis Rats by Alleviating Neuroinflammation

**DOI:** 10.1155/2023/8887091

**Published:** 2023-11-14

**Authors:** Qiongqiong Gao, Zhentao Gao, Minzhi Su, Yong Huang, Chi Zhang, Cuiping Li, Hailun Zhan, Bolong Liu, Xiangfu Zhou

**Affiliations:** ^1^Department of Urology, The Third Affiliated Hospital and Lingnan Hospital of the Sun Yat-Sen University, 2693 Kaichuang Road, Guangzhou 510700, China; ^2^Department of Rehabilitation, The Third Affiliated Hospital and Lingnan Hospital of the Sun Yat-Sen University, 2693 Kaichuang Road, Guangzhou 510700, China; ^3^Department of Biotherapy Center, The Third Affiliated Hospital and Lingnan Hospital of the Sun Yat-Sen University, 2693 Kaichuang Road, Guangzhou 510700, China

## Abstract

Interstitial cystitis/bladder pain syndrome (IC/BPS) seriously reduces the patient's quality of life, yet current therapies only provide partial relief. In the spinal dorsal horn (SDH), neuroinflammation plays a pivotal role in the development of IC. Injection of human umbilical cord mesenchymal stem cells (hUMSCs) to reduce inflammation is an effective strategy, and heme oxygenase-1 (HO-1) exhibits anti-nociceptive effect in neuroinflammatory pain. This study aimed to test the therapeutic effects of hUMSCs overexpressing HO-1 on cyclophosphamide-induced cystitis rat model. Cystitis rats were transplanted with altered cells and then assessed for 3 weeks. A series of behavioral measurements would be trial including suprapubic mechanical allodynia, depressive-like behaviors, micturition frequency, and short-term memory function. Additionally, western blot, immunofluorescence staining, and ELISA kit test for anti-inflammation effect. HUMSCs were capable of being transduced to overexpress HO-1. Injection of hUMSCs overexpressing HO-1 was more effective than hUMSCs alone in alleviating behavioral symptoms in rats. Furthermore, hUMSCs overexpressing HO-1 inhibited the activation of glial and TLR4/p65/NLRP3 pathway, decreased the levels of pro-inflammatory cytokines in the SDH region. Surprisingly, it markedly increased anti-inflammatory cytokine IL-10, reduced MDA content, and protected GSH concentrations in local environment. Our results suggest that injecting hUMSCs overexpressing HO-1 intrathecally can significantly promote functional outcomes in cystitis rats by reducing neuroinflammation, at least, partly through downregulating TLR4/p65/NLRP3 signaling pathway in the SDH region. This cell therapy affords a new strategy for IC/BPS treatment.

## 1. Introduction

Interstitial cystitis/bladder pain syndrome (IC/BPS), which is characterized by bladder pain with nocturia, urgency, nocturia, and sterile urine, is an enigmatic chronic inflammatory disease of the bladder [1–3]. Women with IC/BPS often accompany with depression and memory problems, which could amplify the symptoms of BPS/IC and seriously affect the patient's quality of life [4, 5]. Although multiple theories have been presented, the pathophysiological mechanisms of IC/BPS are poorly understood, and current therapies for IC/BPS are only providing partial relief [6]. Therefore, a new effective therapy to treat IC/BPS is highly demanded.

Our previous studies showed that neuroinflammation in the spinal dorsal horn (SDH) contribute to bladder hypersensitivity in the pathogenesis of IC/BPS [7–9]. During this progress, the TLR4/p65/NLRP3 pathway in neurons is activated to produce inflammatory factors [10], which is accompanied with oxidative stress, further aggravating inflammatory damage [11–13]. Moreover, microglia and astrocytes are activated to produce pro-inflammatory cytokines via the p38/JNK signaling pathway, which can drive and maintain central sensitization [9]. Therefore, treating neuroinflammation in IC/BPS may be a useful strategy.

Heme oxygenase-1 (HO-1, encoded by HMOX1) is a rate-limiting enzyme that catalyzes the heme into biliverdin, carbon monoxide (CO) and iron [14]. Due to its potent antioxidant properties and the ability to negatively regulate inflammation, HO-1 has been used in a variety of inflammatory diseases. Accumulating evidence has demonstrated that anti-nociceptive effect of HO-1 in both acute and chronic inflammatory pain: the peripheral upregulation of HO-1 reduces the inflammatory pain induced by peripheral injection of formalin [15], interleukin-1*β* (IL-1*β*) [16], or complete Freund's adjuvant [17]. Pharmacological activation of HO-1 also prevents neuropathic pain caused by sciatic nerve chronic constriction injury, and reduced the expression of pro-inflammatory cytokines in the spinal cord [18]. Moreover, HO-1 induced by expressing lentivirus can inhibit glial activation through reducing activation of MAPKs in vincristine induced neuropathic pain [19]. The encouraging results of these studies enlighten us that HO-1 may alleviate neuropathic pain in IC/BPS.

Mesenchymal stem cells (MSCs) also possess potent anti-inflammatory and immunomodulatory activities proven by the researches [20], and stem cell therapy based on MSCs is considered a promising alternative for diseases associated with multiple inflammations [21, 22]. Cause MSCs have the high ability of genetic engineering, transplantation of altered cells has been widespread applied to get better therapeutic effects. Due to the stimulated expression of anti-inflammatory cytokines, studies have demonstrated that MSCs modified by HO-1 gene can improve healing [23], enhance survival after engraftment, and confer a more potent protective effect in heart disease [24]. This poly therapeutic strategy of genetically engineered MSCs with HO-1 has been shown to reduce inflammation in collagen-induced model of arthritis [25], spinal cord injury (SCI) [26], and ischemia–reperfusion injury in the kidney [27]. Based on these studies, we hypothesized that the genetically engineered MSCs with HO-1 may resolve neuroinflammation in IC/BPS.

Thus, this study was to investigate if treatment with MSCs overexpressing HO-1 could reduce inflammation and relieve symptoms of IC/BPS. Considering the possibility of practical application, here we used human umbilical cord mesenchymal stem cells (hUMSCs) which are easier to obtain, and have lower immunogenicity than other types of hMSCs [28]. In order to effectively imitate the IC/BPS, referring to a previous literature, we used adult female Sprague–Dawley rats to construct chronic painful bladder model by intermittent intraperitoneal injection of cyclophosphamide (CYP). Mechanical threshold, behavioral experiment, activation of glial cells, levels of inflammatory cytokines, and inflammasomes were examined to assess the therapeutic effect of hUMSCs overexpression HO-1 in CYP-induced cystitis rat model.

## 2. Materials and Methods

### 2.1. Cell Culture

The hUMSCs were provided by the Biotherapy Center, the Third Affiliated Hospital and Lingnan Hospital of the Sun Yat-Sen University (Guangzhou, Guangdong). The cells were cultured with Dulbecco's modified Eagle's medium (DMEM) supplemented with 10% fetal bovine serum (FBS), 100 *μ*g/ml streptomycin, and 100 U/ml penicillin (all from Gibco, USA), in 5% CO_2_ at 37°C. The medium was replaced every 3 days.

For osteogenic differentiation and adipocyte differentiation, hUMSCs culture with osteogenic differentiation medium containing alpha-Minimum Essential Medium supplemented with 500 *μ*M L-ascorbic acid, 5% FBS, 10 mM *β*-glycerophosphate, 100 nM dexamethasone, and 1% penicillin/streptomycin, or adipogenic differentiation medium comprising 1 *μ*M dexamethasone, 0.5 *μ*M 3-isobutyl-1-methyl-xanthine, 10 *μ*g/ml insulin, 200 *μ*M indomethacin, and 1% penicillin/streptomycin in DMEM-low glucose supplemented with glutamine and 10% FBS (all from Gibco, USA) for 22 days. After stained with Alizarin red S or oil Red O, the cells were observed by using a light microscope (Leica DMI4000B,). Measured resolution of 1,024 lp/mm with no downstream processing or averaging that enhances the resolution of image. [29–31].

### 2.2. Modification of HO-1 Gene in hUMSCs via Lentiviral Transduction

Lentivirus transductions without (hUMSCs) or with HO-1 gene (HO-1-hUMSCs) were conducted according to the previously established method with small modifications. In brief, hUMSCs were incubated with lentivirus at the MOI of 20 for 24 hr at 37°C. The transfection activities hUMSCs were observed by the expression of GFP through a fluorescent microscope after 2 days, then puromycin (0.5 *μ*g/ml) was used to remove uninfected cells.

### 2.3. Flow Cytometry

CD34 and CD45 or CD90 and CD73 surface cell membrane markers (all from EXbio, Czech Republic) were used for identing cultured cells. Using A FACS Calibur Flow Cytometer (BD Biosciences, San Jose, USA) to count cells, and the FlowJo software (version 10.1) was applied for data analysis.

### 2.4. Western Blotting (WB)

In vitro experiment part, cells were collected and lysed in 1.5 ml EP tube, the bicinchoninic acid (BCA) protein assay was used to determine the protein concentration. Using sodium dodecyl sulfate polyacrylamide gel (10%) electrophoresis to separated protein samples and then transferred onto polyvinylidene fluoride membranes. The membranes were incubated at 4°C overnight with the primary antibodies: HO-1(1 : 1,000, NB100-98682; Novus Biological), GAPDH (1 : 5,000, T0004; Affinity Biosciences), then incubated with the secondary antibodies for 1 hr at room temperature. Enhanced Chemiluminescence (ECL) kit was used to detect protein bands, and the band density was quantified via a computer-assisted imaging analysis system (ImageJ).

In vivo experiment part, rats were executed with carbon dioxide and the L6-S1 SDH was separated and immediately stored at −80°C for further analysis. The rest of the steps are the same as in the in vivo experiment part. The primary antibodies: TLR4 (1 : 1,000, AF7017; Affinity Biosciences), p65 (1 : 1,000, ab16502; Abcam), phospho-p65 (*p*-p65, 1 : 1,000, AF3389; Affinity Biosciences), NLRP3 (1 : 1,000, NBP2-12446; Novus Biologicals), ionized calcium-binding adapter molecule 1 (Iba-1, 1 : 1,000, ab5076; Abcam), glial fibrillary acidic protein (GFAP, 1 : 1,000, #3670; Cell Signaling Technology), phosphor-p38 (*p*-p38,1 : 1,000, #4511; Cell Signaling Technology), phosphor-JNK (*p*-JNK, 1 : 1,000, #9251s; Cell Signaling Technology), IL-10 (1 : 1,000, ab133575; Abcam), and *β*-actin (1 : 1,000, #3700; Cell Signaling Technology).

### 2.5. Animal Modeling

All experiments were approved by the Institutional Animal Care and Use Committee of Sun Yat-sen University (NO: SYSU-IACUC-2022-000488) and conducted in accordance with the guidelines of the National Institutes of Health on animal care and ethical guidelines. Adult female Sprague–Dawley rats with a weight of 200–220 g were purchased from the Institute of Experimental Animals at the Sun Yat-Sen University and been observed 7 days for quarantine. All rats were tested for Von Frey filaments test, rats with withdrawal value lower than 0.8 were excluded from the experiment. The rats were numbered and randomly assigned to each group with the random sequence generated by EXCEL, and housed in the separate licensed animal units at a temperature of 24°C and 12 hr day/night cycle. Food and water were always available and libitum. CYP (50 mg/kg; Sigma) was systemic injected intraperitoneally (i.p.) to establish the chronic cystitis rat model [32]. On the 8 days after the first CYP injection, rats with withdrawal value higher than 25 were also excluded from the experiment to ensure successful establishment model. At least two operators used double-blinded strategy for each operation to minimize potential confounders. All the rats were executed with carbon dioxide after the completion of the experiment at 21 days. During the whole experiment period, the status of rats was monitored every other day, and euthanasia was timely carried out in case of agonal conditions such as dyskinesia and dyspnea.

### 2.6. Study Design and Animal Groups

The experimental unit is single animal. In order to test the therapeutic effect of transfected MSCs on IC rats, we divided the rats into four groups: the control group, which was injected intraperitoneally with 0.9% normal saline and sterile PBS i.t., the CYP group, which was injected with CYP as previously and sterile PBS i.t., the hUMSC group, which was injected with CYP and hUMSCS i.t., and the HO-1-hMSC group, which was injected with CYP and HO-1-hMSCs i.t.. The number of cells injected into each rat was 1 × 10^6^. According to the preexperiment results, at least five rats are required for each group [10, 11, 33, 34].

To reduce suffering, the rats for urodynamic test were executed by carbon dioxide after the test, and no subsequent experiment was carried out. The rats receiving SPT and NORT were subjected to Von Frey filaments test at the same time. All experiments were repeated four times to ensure adequate tissue materials for western blotting, MDA, GSH, immunofluorescence analysis, filling cystometry, and to obtain statistical significance. The total number of rats used in this experiment is 103.

### 2.7. Intrathecal Injection

The rats were anesthetized and unconscious by 2% isoflurane mixed with 50% oxygen in nitrogen, intrathecal cell delivery procedure has been described in detail by Zhang et al. [10]. A flicking of the tail was considered as a successful puncture.

For cell localization assays, the cells were labeled by fluorescent dye Dil (C7000, Invitrogen™, USA) before transplantation. Three days after cell transplantation, the animal was anesthetized and unconscious by 20% (*w*/*v*) urethane (1 g/kg, intraperitoneal injection), and perfused with 100 ml of cold 0.9% saline then followed by 150 ml paraformaldehyde 4%. Transverse paraffinized sections were 10 *μ*m and stained with 4′, 6-diamidino-2-phenylindole (DAPI). Images were performed as same as stated in immunofluorescence. To validate the cell delivery method, this experiment was done on three animals.

### 2.8. Von Frey Filaments Test

The Von Frey filaments test was used to assessed withdrawal threshold which has been described before detailly [32]. A positive behavioral response includes licking, scratching the area of stimulation, jumping up quickly, and arising. Behavioral measurement performed first, when CYP injections and this test conducted on the same day.

### 2.9. Filling Cystometry

As the filling cystometry requires the rats to remain stationary for more than an hour, and accompanied by pain, 20% (*w*/*v*) urethane (1 g/kg, intraperitoneal injection) was used to anesthetize the rats and make them unconscious. The operation of the filling cystometry was has been described before detailly, and we using BL New Century 2.1 software (Taimeng Technology, China) to record the continuous urodynamic curves [33]. After voiding cycles stabilized, an additional 30 min was recorded for the quantitative analysis.

### 2.10. Behavioral Experiments

To determine depressive-like behaviors and short-term memory ability, sucrose preference test (SPT), and novel object recognition test (NORT) were also carried out, those tests were performed and data points during the analysis as previously described in the same [9].

### 2.11. Immunofluorescence

The excised spinal cord obtained as same as in the cell localization assays. The tissue was cut into 15 *μ*m-thick sections, the primary antibodies: NLRP3 (1 : 100, NBP2-12446; Novus Biologicals), OX-42 (1 : 500, ab1211; Abcam), and GFAP (1 : 400, #3670; Cell Signaling Technology), the secondary antibodies conjugated with Cy3. Measuring and imaging were performed by a Leica fluorescence microscope (Carl Zeiss Microscopy GmbH). We used the same exposure, gain, and gamma every time to keep the images standardized. Measured resolution of 2,136 × 2,136 with no downstream processing or averaging that enhances the resolution of image. ImageJ was also used to quantify the fluorescent intensity of each image.

### 2.12. Enzyme Linked Immunosorbent Assay (ELISA)

To detect the levels of TNF-*α* and IL-1*β*, we used ELISA kits (Jiancheng Biotech, China) according to the manufacturer's instructions. The tissue came from the same rats in WB experiment. The absorbance value was measured by microplate reader at 450 nm.

### 2.13. Glutathione (GSH) Assay and Malondialdehyde (MDA) Assay

The concentration of GSH and MDA were detected by GSH assay kit and MDA Assay Kit (Beyotime, China). The tissue came from the same rats in WB experiment. The absorbance value was measured by microplate reader at 450 nm (for GSH) and 535 nm (for MDA).

### 2.14. Statistical Analysis

SPSS 23.0 software was used for all the statistical analyses. All data were expressed as the mean ± standard error of the mean (SEM). The Shapiro–Wilk test was used to verify normal distribution of the data before each ANOVA test. Data from the SPT, NORT, western blot, and the immunofluorescence assay were analyzed with a one-way analysis of ANOVA followed by Turkey test. When analyze the data of the mechanical threshold, a repeated-measure two-way ANOVA followed by a Turkey test was used. A probability level of *p* ≤ 0.05 was considered statistically significant.

## 3. Results

### 3.1. Morphology and Immunophenotypic Characteristics of hUMSCs

**Figure 1:**
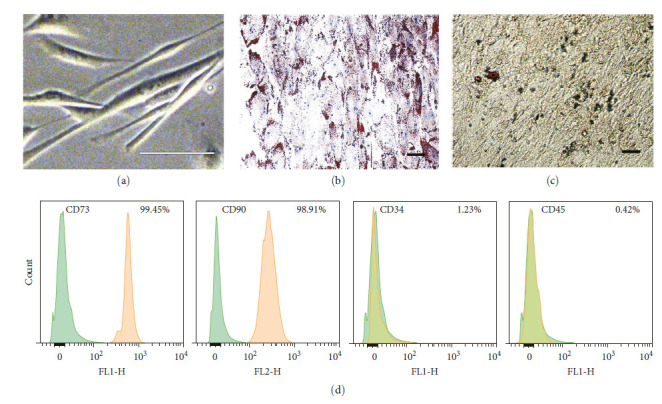
Characterization of hUMSCs. (a) Morphological observation of hUMSCs used in this study. (b) Oil Red O staining was used to evaluate adipogenic differentiation. (c) Alizarin red S staining was used to evaluate osteogenic differentiation. (d) Flow cytometric analysis of hUMSCs surface markers. The cells were positive for CD90 and CD73, and negative for CD45 and CD34. Scale bar: 20 *μ*m.

A fibroblast-like morphology was exhibited by the cultured cells (Figure 1(a)). The osteogenic-induced hUMSCs (stained with Alizarin red S) and adipogenic-induced hUMSCs (stained with oil red O) confirmed the multipotential differentiation capacity (Figures 1(b) and 1(c)). The results of flow cytometry showed that hUMSCs were strongly positive for CD73 = 99.45% and CD90 = 98.91%, negative for CD34 = 1.23% and CD45 = 0.42% (Figure 1(d)).

### 3.2. Efficient Transduction of Human HO-1 in hUMSCs

**Figure 2:**
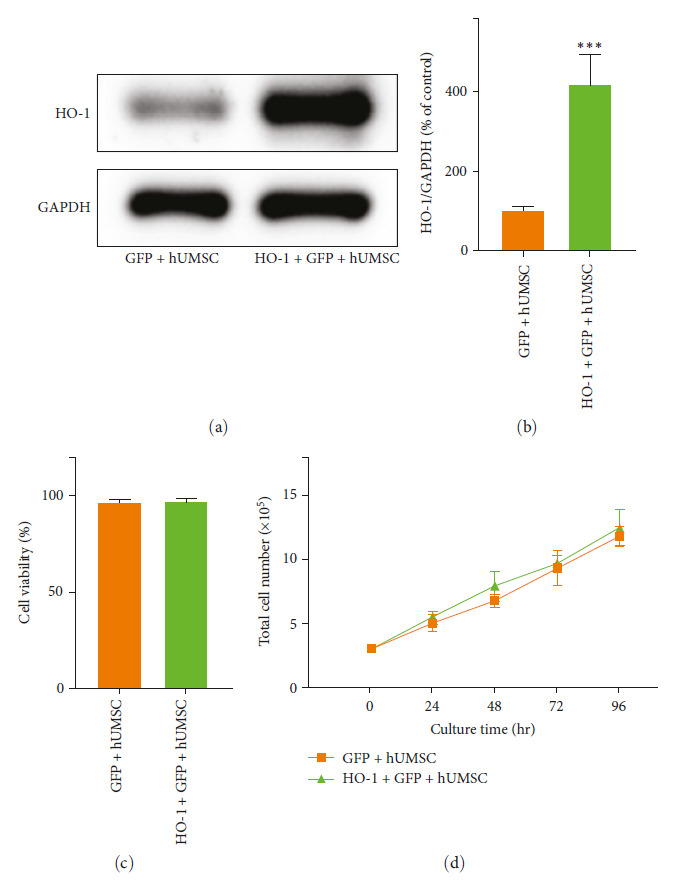
In vitro expression of HO-1 in transduced hUMSCs. (a, b) Western blot HO-1 level was compared between the two groups. (c) Cell viability assay. (d) Time course of cell proliferation.  ^*∗*^*p* < 0.05 vs. the GFP + hUMSC group. Error bars represent standard error of the mean (±SEM).

The hUMSCs were transduced with commercially available lentivirus vectors, and we affirmed that the protein level of HO-1 was upregulated after transduction by WB results (Figures 2(a) and 2(b)). No significant differences in cell viability (Figure 2(c)) and doubling time were detected between groups after gene transfer (Figure 2(d)).

### 3.3. HO-1-hUMSCs Alleviate the Mechanical Allodynia in CYP-Induced Cystitis Rats

**Figure 3:**
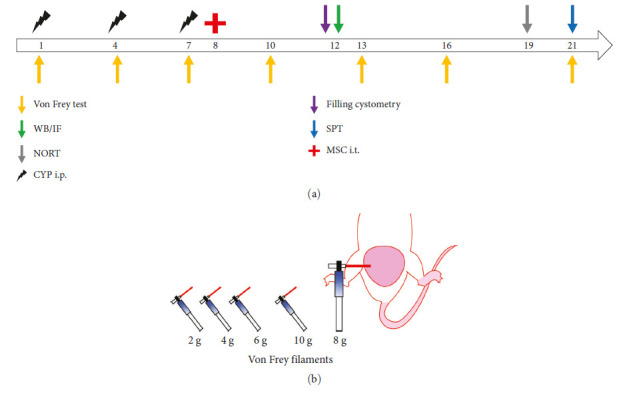
Flowchart of the experimental procedure and schematic illustration of the Von Frey filaments test. (a) Rats were injected with CYP on Days 1, 4, and 7, and received different treatments on Day 8. The effects of treatment were determined by behavioral tests and molecular experiments. (b) The filaments were applied to the suprapubic region with a variety of Von Frey filaments (2, 4, 6, 8, 10, and 15 g). The positive behavioral responses were recorded, including writhing or lifting the body, licking, or scratching the lower abdominal area, and jumping up.

**Figure 4:**
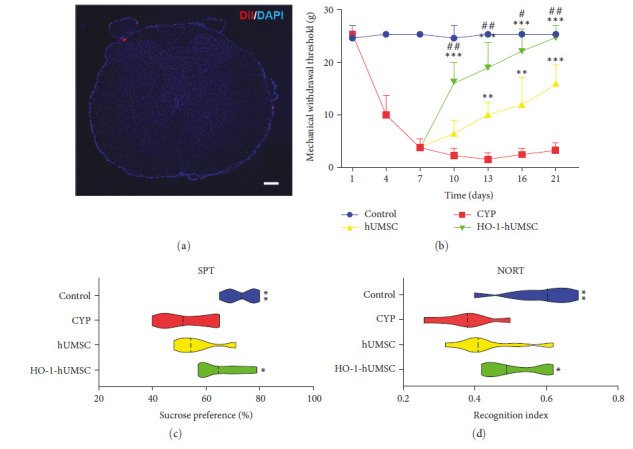
Transduced hUMSCs attenuates mechanical allodynia and comorbid behaviors via paracrine. (a) hUMSCs were labeled with Dil (red), and the fluorescent section showed that the cells failed to enter the spinal cord parenchyma, suggesting that hUMSCs treat inflammation through paracrine (bar = 200 *μ*m). (b) Changes in the mechanical allodynia threshold after treatments (*n* = 10 per group). The data were analyzed by two-way ANOVA followed by the Tukey post hoc test. (c) Sucrose preference test (SPT) were performed on Day 21 (*n* = 8 per group). The data were analyzed by one-way ANOVA followed by the Tukey post hoc test. (d) Novel object recognition test (NORT) was performed on day 19 (*n* = 8 per group). The data were analyzed by one-way ANOVA followed by the Tukey post hoc test.  ^*∗*^*p* < 0.05,  ^*∗∗*^*p* < 0.01, and  ^*∗∗∗*^*p* < 0.001 vs. the control group, and ^#^*p* < 0.05 and ^##^*p* < 0.01 vs. the CYP group. Error bars represent standard error of the mean (±SEM).

For animal experiments, our reporting adheres to the ARRIVE guidelines. The experimental procedure is shown in Figure 3(a). The chronic cystitis rat model was induced by CYP in 7 days and accordingly confirmed by Von Frey filaments test with positive responses (Figure 3(b)). No adverse reaction or rejection was observed in all rats injected with cells. The hUMSCs were labeled with Dil (red), and the fluorescent section showed that the cells do not enter the spinal cord parenchyma (Figure 4(a)), suggesting that hUMSCs treat inflammation through paracrine. The mechanical withdrawal threshold was recorded to investigate the therapeutic efficacy of hUMSCs or HO-1-hUMSCs. There was a significant difference between CYP group and the hUMSC or HO-1-hUMSC group on Day 13, Day 16 and Day 21(Figure 4(b)). Remarkably, HO-1-hUMSC group significantly raised suprapubic mechanical withdrawal threshold from Day 10.

### 3.4. HO-1-hUMSCs Promote Recovery of the Comorbid Depressive-Like Behaviors and Memory Deficits

To more comprehensively assess the effects of cell therapy on animal behavior, we examined cognitive behavioral experiments and performed sucrose preference experiments. The HO-1-hUMSC group preferred sucrose more than the CYP groups (Figure 4(c)) and showed better memory ability as well (Figure 4(d)). But there were no statistically significant differences between the CYP group and the hUMSC group in the above two experiments.

### 3.5. HO-1-hUMSCs Improve the Bladder Micturition Function in CYP-Induced Cystitis Rats

**Figure 5:**
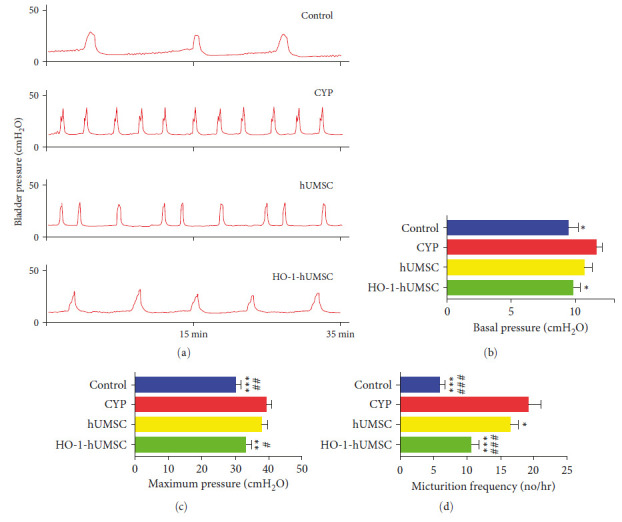
Transduced hUMSCs improve bladder micturition function in CYP-induced cystitis rats. (a) Representative conscious cystometry results in different groups. (b–d) The statistical analysis of the basal pressure, maximum pressure, and micturition frequency (*n* = 7 per group)  ^*∗*^*p* < 0.05,  ^*∗*^ ^*∗*^*p* < 0.01, and  ^*∗*^ ^*∗*^ ^*∗*^*p* < 0.001 vs. the CYP group, and ^#^*p* < 0.05, ^##^*p* < 0.01, and ^###^*p* < 0.001 vs. the hUMSC group. Error bars represent standard error of the mean (±SEM).

Urodynamics was also performed to evaluate the treatment effect of bladder micturition function. As shown in Figure 5(a)–5(d), the CYP-induced cystitis rats exerted irregular voiding patterns, and the maximum pressure in the CYP group was significantly higher than the control group and the HO-1-hUMSC group. The HO-1-hUMSC group exhibited obviously lower basal pressure while the CYP group had the highest basal pressure. Furthermore, compared with the CYP group, the micturition frequency was significantly lower in HO-1-hUMSC group. Above results indicated that HO-1-hUMSCs could promote the improvement of urination parameters in CYP-induced cystitis rats.

### 3.6. HO-1-hUMSCs Restrain the Activation of Astrocytes and Microglia in the SDH Region of CYP-Induced Cystitis Rats

**Figure 6:**
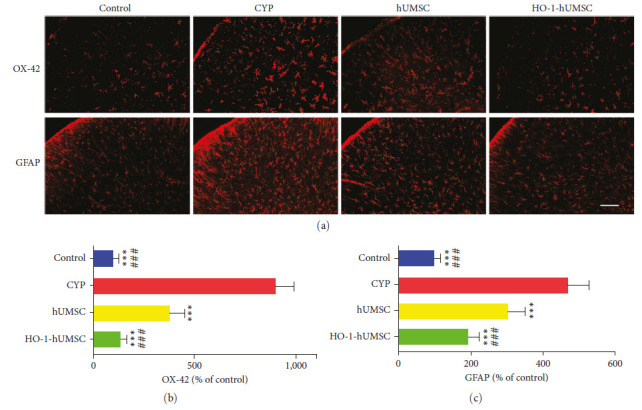
Immunofluorescence staining shows that transduced hUMSCs inhibit the activation of glial cell. (a) Immunofluorescence staining of OX-42 (red) and GFAP (red). Scale bar = 100 *μ*m. (b, c) The statistical charts show the percent of fluorescence intensity in each group. All data were calculated as mean (±SEM) (*n* = 4 per group).  ^*∗*^ ^*∗*^ ^*∗*^*p* < 0.001 vs. the CYP group, and ^###^*p* < 0.001 vs. the hUMSC group.

**Figure 7:**
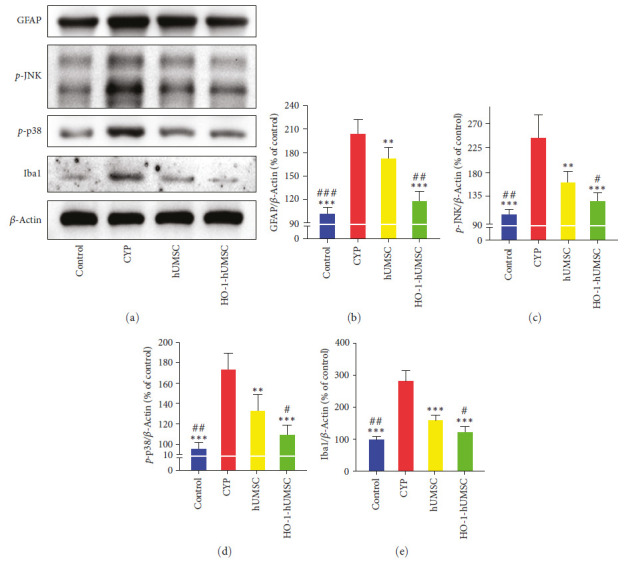
Western blot shows that transduced hUMSCs inhibit the activation of glial cell. (a–e) Western blot analysis showing that intrathecal injection of HO-1-hUMSCs significantly decreased expression levels of GFAP, *p*-JNK, *p*-p38, and Iba1 in the SDH of CYP-induced cystitis. All data were calculated as mean (±SEM) (*n* = 6 per group).  ^*∗*^ ^*∗*^*p* < 0.01,  ^*∗*^ ^*∗*^ ^*∗*^*p* < 0.001 vs. the CYP group, and ^#^*p* < 0.05, ^##^*p* < 0.01, and ^###^*p* < 0.001 vs. the hUMSC group.

In order to explore the effect of our cell therapy on the neuroinflammation, we used immunofluorescence staining (Figure 6(a)–6(c)) and WB (Figure 7(a)–7(e)) to detect the degree of activation of microglia and astrocytes in the SDH region. The main activation pathway of glial cells is p38/JNK. The results showed that the expression of OX42/IBA-1 (microglia) and GFAP (astrocyte) were significantly reduced after cell therapy, and the results of WB also supported that treatment with HO-1-hUMSCs was effective in inhibiting glial cells activation through reducing the content of *p*-p38 and *p*-JNK.

### 3.7. HO-1-hUMSCs Downregulate TLR4/p65/NLRP3 Signal Pathway in the SDH Region of CYP-Induced Cystitis Rats

**Figure 8:**
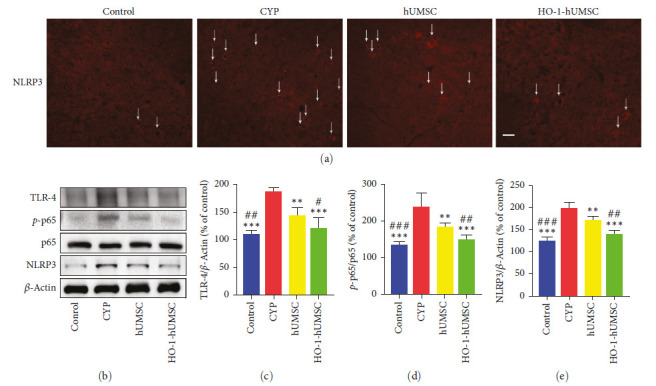
Transduced hUMSCs inhibit activity of TLR4/p65/NLRP3 signal pathway in SDH region. (a) Immunofluorescence staining of NLRP3 (red), the arrows were pointing to NLRP3 positive cells. Scale bar = 50 *μ*m. (b–e) Western blot analysis showing that expression level of TLR4, phosphorylation ratio of p65, and NLRP3 were significantly decreased in SDH after HO-1-hUMSCs treatment. All data were calculated as mean (±SEM) (*n* = 6 per group).  ^*∗*^ ^*∗*^*p* < 0.01,  ^*∗*^ ^*∗*^ ^*∗*^*p* < 0.001 vs. the CYP group, and ^#^*p* < 0.05, ^##^*p* < 0.01, and ^###^*p* < 0.001 vs. the hUMSC group.

In our previous research, we have proved that the TLR4/p65/NLRP3 signal pathway was been excessively activated, and played a key role in the spinal neuroinflammation in CYP-induced cystitis rats [10]. Our results also confirm this. After injected with CYP, the expression level of TLR4, phosphorylation ratio of p65, and NLRP3 were significantly increased. The results of immunofluorescence staining also showed that more NLRP3 positive cells were observed in the CYP group (Figure 8(a)). To further verified whether TLR4/p65/NLRP3 signal pathway was inhibited after therapy by HO-1-hUMSCs in CYP-induced cystitis rats, western blot analysis was been applied. As shown in Figure 8(b)–8(e), the expression levels of TLR4, *p*-p65, and NLRP3 were significantly reduced in HO-1-hUMSC group compared to the CYP group. Based on the above data, we concluded that HO-1-hUMSCs could inhibit the activity of TLR4/p65/NLRP3 signal pathway in the SDH of CYP-induced cystitis rats.

### 3.8. HO-1-hUMSCs Reduce the Level of Inflammatory Factors and Conservate Antioxidant Level in the SDH Region of CYP-Induced Cystitis Rats

**Figure 9:**
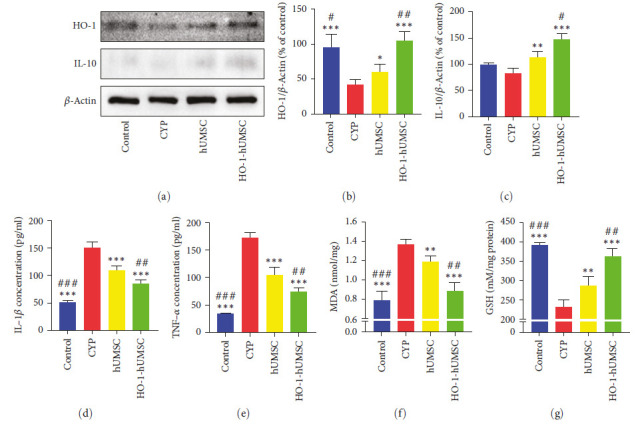
The level of cytokines and oxidative stress in SDH. (a–c) Western blot analysis showing that expression level of anti-inflammatory cytokine IL-10 and the cytoprotective protein HO-1 were significantly increased after HO-1-hUMSCs treatment. (d, e) ELISA showing that the pro-inflammatory cytokines IL-1*β* and TNF-*α* were significantly decreased after HO-1-hUMSCs treatment. (f, g) MDA, GSH levels were detected in the SDH of different groups. All data were calculated as mean (±SEM) (*n* = 6 per group).  ^*∗*^*p* < 0.05,  ^*∗*^ ^*∗*^*p* < 0.01, and  ^*∗*^ ^*∗*^ ^*∗*^*p* < 0.001 vs. the CYP group, and ^#^*p* < 0.05, ^##^*p* < 0.01, and ^###^*p* < 0.001 vs. the hUMSC group.

The level of inflammatory factors in the SDH area had also been detected. After treating with HO-1-hUMSCs, pro-inflammatory factors IL-1*β* and TNF-*α* were significantly decreased. It is worth noting that hUMSCs can also increase the content of HO-1 and IL-10 in the SDH region (Figure 9(a)–9(e)). Considering the ability of HO-1 to resist oxidative stress, we used kits to detect the contents of oxidative stress markers MDA and GSH in the SHD region (Figures 9(f) and 9(g)). Surprisingly, HO-1 can significantly reduce MDA content and protect GSH concentration in this region.

## 4. Discussion

In this study, we demonstrated that hUMSCs were capable of being transduced to overexpress HO-1 in vitro and in vivo. In vivo treatment with hUMSCs alone could alleviate suprapubic mechanical allodynia and frequent micturition in CYP-induced cystitis rats, while hUMSCs overexpressing HO-1 had better therapeutic effect. Additionally, rats treated with HO-1-hUMSCs relieved the comorbid depressive-like behaviors and memory deficits. In the SDH region, HO-1-hUMSCs treatment showed significant advantages over hUMSCs in inhibiting the activation of glial, decreasing the level of pro-inflammatory cytokine through downregulating the TLR4/p65/NLRP3 pathway, and HO-1-hUMSCs markedly increased the level of anti-inflammatory factor IL-10, reducing the MDA content and protecting the GSH concentration in CYP-induced cystitis rats at the same time. These findings verified our hypothesis that intrathecal injection of hUMSCs overexpressing HO-1 can promote functional outcomes in cystitis rats through reducing neuroinflammation, and the TLR4/p65/NLRP3 pathway may be the potential regulatory target.

Numerous researches indicated that MSCs enter the target region through local seeding or blood flow and can contact with surrounding cells in animal models such as spinal cord injury or ischemia–reperfusion. However, in our CYP-induced cystitis rats, the hUMSCs localized to the pia mater and did not enter the spinal cord parenchyma, which was the same as previous experiment [34]. It is speculated that in our model, hUMSCs modulate inflammation through paracrine, rather than direct recognition. Considering the blood–brain barrier, intrathecal injection is more effective treatment for neuroinflammation. Our results indicated that injecting hUMSCs or HO-1-hUMSCs intrathecally can inhibit the activation of glial cells, reduce the level of pro-inflammatory cytokine in the SDH, and alleviate the disturbance of the urination reflex controlled by the lower control center, which may close to the SDH region [35]. Surprisingly, attenuation of depressive-like symptoms, and memory deficits were also observed, which is linked to neuroinflammation in hippocampus, prefrontal cortex, and amygdala [36–38]. Thus, we believe that intrathecal injection of HO-1-hUMSCs can help to alleviate neuroinflammation in the central nervous system in our CYP-induced cystitis rats.

Our previous study showed that the activation of NLRP3 plays an important role in the CYP-induced cystitis rats, and intrathecal injected hUMSC-derived extracellular vesicles can restrain activation of TLR4/p65/NLRP3, attenuate neuroinflammation in the SDH region [10], which is as same as this that hUMSCs along can suppressing activation of TLR4/p65/NLRP3 pathway. Our previous studies also showed that microglia and astrocytes are activated to produce pro-inflammatory cytokines TNF-*α* via the p38/JNK signaling pathway [9], and this signaling pathway can be inhibited by hUMSC-derived extracellular vesicles [10] or exogenous induction of HO-1 [19]. Furthermore, other studies have shown that HO-1 can inhibit the activation of microglia by downregulating the TLR4/p65 signaling pathway [39–42], thereby reducing the levels of IL-1*β* and TNF-*α* in chronic mild stress-induced depressive-like behaviors [39]. Our result agreement with that, compared with hUMSCs, HO-1-hUMSCs can more significantly inhibit glial cell activation and reduce the level of pro-inflammatory factors in SDH region.

There is now evidence that the relationship between oxidative stress and TLR4/p65/NLRP3 signal pathway is very close. First, TLR4/p65 could mediate ROS production. A previous experiment demonstrated that ROS production induced by Ang-II was almost completely absent in TLR4-deficient compared with TLR4-sufficient mice, indicating that TLR4 plays a key role in mediating ROS production [11]. Second, oxidative stress could induce TLR4 expression. In other acute lung injury diseases, increased activation of TLR4 can lead to the induction of oxidative stress, while the oxidation of phospholipids causes TLR4 sensing ROS production and increasing TLR4 expression [12]. Third, ROS could be involved in NLRP3 priming and activation. One example of this is crystal. When exogenous asbestos interacting with cells, the produced ROS, in turn, could lead to the generation of ligand of NLRP3 or alternatively modify NLRP3 directly [43]. In this experiment, we are very pleased to see that the indicators related to oxidative stress such as MDA and GSH are significantly improved after treatment, which is consistent with the trend of TLR4/p65/NLRP3 pathway. The use of MSCs overexpressing HO-1 has already been applied in many animal models, and it has been declared that HO-1 can protect the viability of transplanted MSCs by effectively alleviating the oxidative stress in the local environment [27, 44, 45]. This valuable multipotent approach inhibits apoptosis and promotes the function of injured tissue better than injecting MSCs alone. Our results showed the same trend: although hUMSCs have a certain therapy by suppressing TLR4/p65/NLRP3 signaling pathway, the HO-1-hUMSC group is more effective in relieving oxidative stress and has better performance on most indicators.

In addition, it was found that hUMSCs and HO-1-hUMSCs can increase the level of IL-10 in SDH of CYP-induced cystitis rats. IL-10 is an anti-inflammatory cytokine that can be secrete by leukocyte, and numerous studies have shown that IL-10 can reduce inflammation through direct inhibition of the pro-inflammatory cytokine TNF-*α* [46], reducing hemoglobin-induced oxidative tissue damage [47], and modulation of macrophage expression in many inflammatory diseases such as spinal cord injury [48, 49]. Due to the potent anti-inflammatory effect of IL-10, many studies have used highly expressed IL-10 cells to treat neuroinflammation [23, 50]. And some other studies claimed that after upregulating the expression of HO-1 in MSCs, the expression of IL-10 in MSCs was also increased [25], which may contribute to reduce inflammation. Although we could not determine whether this IL-10 is secreted by glial cells after HO-1-hUMSC treatment, its effect of anti-inflammatory in the SDH region is undeniable.

## 5. Conclusion

Collectively, these results suggest that intrathecal injection of hUMSCs overexpressing HO-1 can significantly promote functional outcomes in cystitis rats by reducing neuroinflammation, at least, partly through downregulating TLR4/p65/NLRP3 pathway in the SDH region of CYP-induced cystitis rats. This work proposes additional support for the content that transplantation of hUMSCs overexpressing HO-1 as a possible new treatment for IC/BPS.

## Data Availability

The datasets supporting the findings of this study are available from the corresponding author on reasonable request.

## Abbreviations

IC: Interstitial cystitis
BPS: Bladder pain syndrome
HO-1: Heme oxygenase-1
SDH: Spinal dorsal horn
MSC: Mesenchymal stem cell
TNF-*α*: Tumor necrosis factor alpha
hUMSCs: Human umbilical cord mesenchymal stem cells
CYP: Cyclophosphamide
GFAP: Glial fibrillary acidic protein
IBA-1: Ionized calcium-binding adapter molecule 1
NLRP3: Nucleotide binding and oligomerization domain-like receptor family pyrin domain-containing 3
IL-1*β*: Interleukin 1 beta
IBA-1: Ionized calcium binding adapter molecule 1
TLR4: Toll-like receptor 4
p65: Nuclear factor kappa B.
